# Reflecting on Psychotherapy Practice for Psychologists: Towards Guidelines for Competencies and Practices

**DOI:** 10.32872/cpe.v2i4.2601

**Published:** 2020-12-23

**Authors:** Anne Plantade-Gipch, Nady Van Broeck, Koen Lowet, Eleni Karayianni, Maria Karekla

**Affiliations:** a School of Practitioners Psychologists, Paris, France; b University of Leuven, Leuven, Belgium; c Flemish Association of Clinical Psychologists, Beringen, Belgium; d University of Cyprus, Nicosia, Cyprus; University of Vienna, Vienna, Austria

**Keywords:** psychotherapy, competencies, specificities of psychotherapy practiced by psychologists, psychologists’ scientific approach to psychotherapy

## Abstract

In 2017, the European Federation of Psychologists Associations made a statement on psychotherapy. It recognizes that psychotherapy is a “special competence” practiced by psychologists, and that psychologists practicing psychotherapy receive specific education, including supervision. The statement also stresses that they have demonstrated competencies in scientifically validated or established theories on human emotions, cognitions, and behavior, and on processes of development, as well as the application of these methods to achieve change. Moreover, the declaration recognizes that they are trained in the scientific application of the methods of change based upon these theories. Within the Standing Committee of Psychology and Health in collaboration with the S-EAC, the group on Psychotherapy is presently working on a conceptual framework and on guidelines for psychotherapy practiced by psychologists. This document is starting to define the necessary skills and competencies for European psychologists practicing psychotherapy. It also makes recommendations for basic training, for the development of practical skills and competencies, for continuing professional development, and for ethical decision making. It especially puts forward psychologists’ scientific approach to psychotherapy.

In Europe, the practice of psychotherapy among psychologists is diverse. As such, it is important to identify the common ground for the work of European psychologists practicing psychotherapy. The European Federation of Psychologists Associations (EFPA) offers the possibility of obtaining a EuroPsy Specialist Certificate in Psychotherapy^1^1The S-EAC-PsyPT is an EFPA body functioning under the EAC (Europsy Awarding Committtee) who is responsible for the granting of the EuroPsy Specialist certificate for Psychologists specialized in Psychotherapy. for psychologists who meet specified requirements. This certificate can be awarded to psychologists - individuals who already possess a basic EuroPsy certificate and who meet the specified training and supervised practice criteria laid out by the specialist certificate. Also, in 2016, a reflection on the skills and competencies of psychologists practicing psychotherapy was launched in France, under the auspices of the French Federation of Psychologists and Psychology. A first working document was developed, which served as a basis to build upon the proposed guidelines. In 2017, during its general assembly, the European Federation of Psychologists Associations (EFPA) created the Psychotherapy group within the Standing Committee of Psychology and Health, which started an exploration around the practices of European psychologists practicing psychotherapy. At the time, the group included representatives from Portugal, Belgium, Cyprus, and France. One of the core principles specified is that the practice of psychotherapy by psychologists is based on scientific evidence. In its two year’s work plan, the group focused on several topics relating to practice, such as skills and competencies, basic training, continuing professional development, and ethical decision making. The current paper presents the work on skills and competencies of this group so far. It is formulated in terms of expectations concerning European psychologists’ practice of psychotherapy. This work will continue to be developed in the years to come.

## Psychotherapy Definition: A Difficult Consensus to Reach

Working on guidelines for psychologists practicing psychotherapy is not simple. Indeed, such a reflection requires a clear definition of psychotherapy. Despite various descriptions of what constitutes psychotherapy provided by both researchers and professional psychologist associations, there is no consensus regarding its definition, especially as uniquely practiced by psychologists. Existing descriptions present a tendency to polarize psychotherapy, either highlighting the active psychological treatment components or emphasizing the therapeutic relationship and encounter. This split is often related to schools of thoughts, whose epistemological foundations diverge, especially from the point of view of human nature and development, which results in different underlying intervention objectives. Also, a chasm often seems to separate research and practice, which is problematic as it hinders achieving one complete and comprehensive definition of psychotherapy (Goldfried et al., 2014). Traditionally, the practice of psychotherapy by psychologists was part of a continuum of care, with, on the one hand, interventions promoting self-exploration, and on the other, therapeutic actions based on counseling principles. For EFPA, the following definition of psychotherapy suggested by Norcross (1990) attained consensus in 2017 between Member Associations: “Psychotherapy is the informed and intentional application of clinical methods and interpersonal stances derived from established psychological principles for the purpose of assisting people to modify their behaviors, cognitions, emotions, and/or other personal characteristics in directions that the participants deem desirable” (EFPA, 2017; Norcross, 1990).

This definition served as a basis for drawing up the initial recommendations made in this paper, concerning the skills expected of psychologists practicing psychotherapy. The practice of psychotherapy in our profession is based on the acquisition and use of established evidence-based psychotherapeutic methods and techniques and scientific knowledge. Psychologists practicing psychotherapy stay informed about the most recent scientific developments concerning methods and techniques, as well as the effectiveness of the therapeutic approaches. The scientific approach to psychotherapy, as practiced by psychologists, is grounded in sound explanatory models of human behavior, emotion, personality, and development. This scientific approach includes the importance of deriving interventions on evidence-based data, as well as adjusting the intervention to the particular situation of the patient, taking into account life contexts, personal characteristics, culture, preferences and values (American Psychological Association [APA], 2006). In this group we believe that scientific investigation of our therapeutic approaches and techniques should be an aim, to ensure that individuals in need receive the best care possible for their problem and circumstances. The practice of psychotherapy by psychologists is overall characterized by a concern for the mental health and overall functioning of the patient, as well as by the rigor of its methods, resulting from scientific data and practical reasoning, as well as based on sound theoretical grounds (EFPA, 2005). The advanced skills and competencies outlined in this paper for psychologists practicing psychotherapy are at their infancy and the work is yet to be finished. In the coming years, this work will be expanded through a project group especially formed by EFPA, and competencies and skills of psychologists practicing psychotherapy will be further developed.

## Psychological Assessment, Diagnosis, and Case Conceptualization Prior to Intervention

Psychologists’ basic training places great importance to the thorough assessment of the mental state, psychopathological symptoms and problems, and the life situation of the patient(s). The practice of psychotherapy by psychologists always includes assessment of functioning and of the patient’s context, for the case conceptualization and the formulation of hypotheses. Assessment can be carried out throughout the psychotherapy process according to patient’s problems and demand (GGZ Standaarden, 2020). Assessment is a specific work methodology of the profession that is supported by the scientific literature (APA, 2006). More specifically, psychologists practicing psychotherapy have extensive knowledge in the field of psychopathology, diagnosis, and its assessment using a variety of methods and tools. Included in their assessment are means to establish a historical account of difficulties and any previous therapeutic attempts (including medical and pharmacological interventions), contributing and maintaining factors, contextual factors, psychopathological symptoms and variables, and comorbid medical and psychological conditions. For these purposes, it is important that psychologists practicing psychotherapy possess a basic knowledge of psychopharmacology as well as the effects of medical problems and treatment on psychopathology. Based on their assessment, they first establish a case conceptualization where they evaluate the psychological and mental state of the patient, the symptoms and level of functioning within context and in relation to social influences and the person's environment. They establish a diagnosis when relevant (e.g., based on the DSM or ICD). They also assess the patients motives and needs for the therapy, their personal and external resources, as well as the subjective and environmental factors that could hinder the intervention. As a prerequisite to the treatment, psychologists practicing psychotherapy build an intervention plan in collaboration with the patient, which then allows both patient and therapist to be able to appraise the therapeutic progress. The case formulation constitutes a working hypothesis, in that based on new knowledge and a continuous assessment process during therapy, changes may be made to it and treatment goals modified accordingly. Progress is also continuously assessed and utilized to further guide the case conceptualization and therapy (Duncan et al., 2010).

## An Expertise in Interviewing and Psychotherapeutic Prevention and Intervention Techniques

Psychologists practicing psychotherapy have an expertise in interviewing techniques, utilizing basic clinical skills, which help to be able to mobilize a patient to talk about their problem. The aim is also to collect valuable information during their assessment. Psychologists practicing psychotherapy consider the patient's right to self-determination, respecting the rules linked to the therapeutic framework, such as, for examples, neutrality and abstinence (EFPA, 2005). Psychologists practicing psychotherapy have a thorough knowledge of intervention techniques belonging to at least one psychological theoretical approach. They utilize a variety of techniques based on the needs of the patient and the context at hand. They work within the limits of a therapeutic framework and a contract, which can be negotiated with the patient.

Based on their theoretical approach, psychologists practicing psychotherapy possess an amalgam of psychotherapeutic prevention and intervention techniques and tools that can be used to achieve the aims and goals for each specific patient. In addition, they possess skills to evaluate the effectiveness of the therapeutic process in a dynamic manner. They make changes in the direction of the intervention as needed, to be able to result in effective behavior change and alleviation of suffering in the patient(s) they serve.

## A Comprehensive Understanding of the Patient's Difficulties

During their basic training, psychologists deepen their knowledge of the major psychological theories of human behavior, development, and psychopathology. Subsequently, they continue to deepen their knowledge in at least one psychotherapeutic theory, grounded in a substantial body of scientific knowledge, and recognized in its applications by the profession of psychologist. Psychologists practicing psychotherapy can justify and explain the interventions they suggest to the patient, based on their case conceptualization and theoretical grounding models. When the situation of the patient requires it and based on their psychotherapy training and their expertise, they may utilize psychological intervention techniques arising from other models than their preferred one, in accordance with the patient’s needs and the empirical literature. They consider the limits of their competence and upon agreement of the patient refer to other professionals or theoretical models when needed (EFPA, 2005). They perceive the difference between the objectives of their own approach, knowledge and expertise, and the needs and demands of the patient. They are aware of the competences of other health care professionals and are trained to collaborate with other health care disciplines when indicated and agreed upon by the client.

## Maintaining a Therapeutic Relationship and an Alliance

Scientific research on evidence-based therapeutic relationships has significantly developed over the last 30 years. Emphasis on the therapeutic relationship heavily influenced the training of practicing psychologists. Psychologists practicing psychotherapy are aware of the factors affecting the therapeutic relationship and their effectiveness as therapists, that allow the patients to reach their therapeutic goals (Barkham et al., 2017). Research demonstrates that certain intrapersonal therapist characteristics (e.g., self-relatedness) may interact with patient characteristics and pathology (Baldwin & Imel, 2013; Heinonen & Nissen-Lie, 2020). These can aid or alternatively hinder the therapeutic process. Psychologists practicing psychotherapy acknowledge these factors and attempt to capitalize on their strengths while aiming to improve on any of their identified weaknesses. They work on taking a step back from the interpersonal difficulties arising in psychotherapy. They give themselves means to analyze these, such as being involved in supervision or peer consultations, or conduct research relating to factors affecting effectiveness (Milne, 2009; Wampold, 2017). They recognize their own contribution to the therapeutic relationship and consider variations in the alliance (Ackerman et al., 2001; Castonguay et al., 2006; Muran & Barber, 2010). Specific assessment techniques can be recommended to assess parameters of the therapeutic relationship and to monitor the patients experience of the therapeutic sessions and process (Gondek et al., 2016).

## Self-Assessment and Professional Help in Difficult Situations

Psychologists practicing psychotherapy evaluate the effects of their interventions as well as the satisfaction of the patient. They recognize their own limits and engage in psychotherapeutic work for which they have demonstrated in depth knowledge and competency. When necessary, they refer to other professionals or approaches having the patients’ needs and well-being as guides. In case of referring out a patient, they ensure a favorable transition to the continuation of treatment (EFPA, 2005).

Continuously learning and updating skills, knowledge, and tools, is an integral part of being a professional. Psychologists practicing psychotherapy are required to demonstrate this with continuing education practices. One area that has entered into the practice of assessment and therapy for which previous generations of therapists had not received training is the digitalization of health and mental health and the availability of new opportunities to utilize technology in therapists’ repertoire of practices (Van Daele et al., 2020). Psychologists practicing psychotherapy should demonstrate flexibility and a drive for new learning and updating.

## Maintaining Patients’ Mental Health and Well-Being at the Center of the Psychotherapy

Psychologists practicing psychotherapy remain aware of the evolving needs of the patient. Their intervention is always carried by their professional ethics. They know their Code of Ethics and how to apply it. They constantly give themselves the means of improving and analyzing situations. The therapeutic process concludes for the benefit of the patients and the consolidation of their therapeutic achievements whilst ensuring that plans are made and resources are known for dealing with difficulties in the future (EFPA, 2005).

## Conclusion

This paper presented an overview of skills and competencies drafted by the EFPA group on Psychotherapy. There seems to be a relative consensus between Member Associations of EFPA that the practice of psychotherapy by psychologists is based on scientific evidence and on practitioners’ expertise. Psychotherapy, its scientific approach, and practice are at the heart of the identity of the profession of psychologists. In the next few years, the Psychotherapy project group of the EFPA Standing Committee on Psychology and Health will enlist experts from all around Europe to tackle issues related to psychotherapy as practiced by psychologists. In collaboration with the EuroPsy Specialist Certificate Awarding Committee, the work of the group will expand particularly from the point of development of competencies, training new professionals, professional parameters, and ethics.
